# Point of No Return—What Is the Threshold of Mitochondria With Permeability Transition in Cells to Trigger Cell Death

**DOI:** 10.1002/jcp.31521

**Published:** 2025-01-06

**Authors:** Kristina A. Kritskaya, Olga A. Stelmashchuk, Andrey Y. Abramov

**Affiliations:** ^1^ Institute of Cell Biophysics of the Russian Academy of Sciences Puschino Russia; ^2^ Orel State University named after I.S. Turgenev Orel Russia; ^3^ Department of Clinical and Movement Neurosciences UCL Queen Square Institute of Neurology, Queen Square London UK

**Keywords:** apoptosis, astrocytes, caspase‐3, fibroblasts, mitochondria, mPTP, necrosis, neurons

## Abstract

Programmed cell death (apoptosis) is essential part of the process of tissue regeneration that also plays role in the mechanism of pathology. The phenomenon of fast and transient permeability of mitochondrial membranes by various triggers, known as permeability transition pore (mPTP) leads to the release of proapoptotic proteins and acts as an initial step in initiation of apoptosis. However, a role for mPTP was also suggested for physiology and it is unclear if there is a threshold in number of mitochondria with mPTP which induces cell death and how this mechanism is regulated in different tissues. Using simultaneous measurements of mitochondrial membrane potential and a fluorescent marker for caspase‐3 activation we studied the number of mitochondria with calcium‐induced mPTP opening necessary for induction of apoptosis in rat primary cortical neurons, astrocytes, fibroblasts, and cancer (BT‐474) cells. The induction of apoptosis was correlated with 80%–90% mitochondrial signal loss in neural cells but only 35% in fibroblasts, and in BT‐474 cancer cells over 90% of mitochondria opens mPTP before apoptosis becomes obvious. The number of mitochondria with mPTP which induce cell death did not correlate with total expression levels of proapoptotic proteins but was consistent with the Bax/Bcl‐2 ratio in these cells. Calcium‐induced mPTP opening increased levels of necrosis which was higher in fibroblasts compared to neurons, astrocytes and BT‐474 cells. Thus, different tissues require specific numbers of mitochondria with PTP opening to induce apoptosis and it correlates to the proapoptotic/antiapoptotic proteins expression ratio in them.

## 1 Introduction

Apoptosis—programmed cell death which plays an essential role in multicellular organisms for tissue renovation and regeneration. Abnormality in the regulation of apoptosis leads to various pathologies including cancer, or in postmitotic neurons, induces neurodegeneration. Apoptosis includes a cascade of reactions which are initiated by the release of proteins from the intermembrane space of mitochondria. This release of proapoptotic proteins from mitochondria is induced by mitochondrial swelling that is triggered by the opening of the mitochondrial permeability transition pore (mPTP). The mitochondrial permeability transition (MPT) refers to a fast and transient alteration in the permeability of the inner mitochondrial membrane and was discovered and intensively studied in the 1950s and 1960s (Azzi and Azzone 1965). In 1979 the main inductor (Ca^2+^) and potential structure that triggers the mitochondrial PT—pore was suggested (Hunter and Haworth 1979). Later, the inhibitor of this structure cyclosporine A (CsA) was identified, and this was the event that paves the way for exploration of the physiological and pathological events connected to mPT (Broekemeier, Dempsey, and Pfeiffer 1989). It should be noted that the structure of mPTP is still actively studied, and it is still a subject of fierce scientific discussion (Bernardi, Carraro, and Lippe 2022). mPTP‐induced mitochondrial swelling leads to the release of mitochondrial proapoptotic factors, such as cytochrome C (Kantrow and Piantadosi 1997), apoptosis‐inducing factor (AIF) (Susin et al. 1999), second mitochondria‐derived activator of caspases (SMAC)/(DIABLO) (Zhou et al. 2005), and endonuclease G (Davies et al. 2003), and any of these factors has been shown to activate apoptosis. The activation of even the initial steps of apoptosis inevitably leads to the death of the cell. Considering this, the first trigger of apoptosis—regulation of the mPTP opening should be vitally important for cells.

There are two major activators of the mitochondrial permeability transition—overload of mitochondria with Ca^2+^ and reactive oxygen species (ROS) overproduction: and it is not surprising that these two activators could also induce cell death. However, not all mitochondria in the cell can induce mPT and not all mPTP opening is associated with cell death (Barsukova et al. 2011; Novikova et al. 2022). Further, physiological relevance of the mPTP opening is still actively discussed (Mnatsakanyan et al. 2017).

Considering this one question arises—how cell distinguishes between physiological and cell death signal when mPTP opens and is there a threshold for the number of mitochondria with mPT to trigger cell death? Therefore, determining the quantitative extent of mPTP‐dependent mitochondrial depolarization needed to irreversibly commit cells to apoptosis is essential for understanding the signaling mechanisms governing cell survival versus death. While neurons have high bioenergetic demand and are postmitotic, rendering them vulnerable to mitochondrial dysfunction, cancer cells evade apoptosis to support uncontrolled growth. Studying apoptotic thresholds across these disparate cell types is essential for drug regulation of cell fate in diseases (Briston et al. 2019). For neurodegenerative conditions, strategies aimed at restoring mitochondrial function may rescue neurons on the brink of apoptosis (Ludtmann et al. 2018). In contrast, chemotherapy that increases mitochondrial vulnerabilities may help force refractory cancer cells to undergo apoptosis (Vazanova et al. 2018).

Here, using simultaneous measurements of mitochondrial membrane potential (ΔΨm) and the fluorescent caspase‐3 substrate NucView‐488 we studied correlation between the number of mitochondria with calcium‐dependent mPTP opening with activation of caspase‐3 in skin fibroblasts, primary neurons and astrocytes and cancer cell line BT‐474. We have found that fibroblasts activate apoptosis after ~30% mitochondria in the cell induce mPT, while in neurons, astrocytes and cancer cells mPTP should be opened when ~90% mitochondria open mPTP to induce cell death. The number of mitochondria with mPTP was correlated with the expression levels of proapoptotic proteins and the Bax/Bcl‐2 ratio. Calcium‐induced mPTP opening increased necrosis which was higher in fibroblasts compared to neurons, astrocytes and BT‐474 cells. Thus, apoptotic cell death is regulated not only by the sensitivity of the cells to mPTP triggers but also by the number of mPTP opening that leads to the release of essential number of proapoptotic proteins.

## 2 Materials and Methods

### 2.1 Primary Rat Neuron‐Glial Culture

The protocol of rat cortical neuron‐glial culture preparation was described in detail earlier (Abramov and Duchen 2010). All animal studies were conducted in accordance with the requirements of the legislation and were approved by the Animal Ethics Committees of the ICB RAS. Wistar rat 1–3 days pups were used for cell culture preparations. The pups were euthanized and decapitated to remove the brain. The cortex was removed into ice‐cold Ca^2+/^Mg^2+^‐free Versene solution (Gibco, UK). After that, the tissue was minced and trypsinized (Gibco, Canada) (0.25% at 37°C) no more than 10 min. Trypsin inactivation was performed by double washing with cold Neurobasal‐A (Gibco, USA). Next, the obtained cell suspension was centrifugated for 3 min at 2000 rpm and the supernatant was carefully removed, and the pellet was resuspended in the growth medium and plated on polyethylenimine‐coated coverslips. For neuron‐glial the growth medium contained Neurobasal‐A, B‐27 supplement (2%), glutamine (0.5 mM) penicillin–streptomycin. Neuron‐glial cultures were maintained at 37°C in a humidified atmosphere of 5% CO_2_ and 95% air, fed twice a week. We used 12–14 DIV (days in vitro) cultures in all experiments.

### 2.2 Human Skin Fibroblasts

Fibroblasts were obtained by biopsy of the donor skin (Kovac et al. 2019). Fibroblasts cells plated on glass coverslips in DMEM (Biological Industries, Kibbutz Beit‐Haemek, Israel) with 10%‐FBS (Biological Industries, Kibbutz Beit‐Haemek, Israel) and 1% GlutaMAX (Gibco, New York, USA) culture medium. The cultures were grown in the incubator for 40%–50% confluence under standard conditions (37°C and 95% humidity). All cells used for experiments were not older than passage 18th.

### 2.3 Cancer Cell Line

Cancer cells (BT‐474) is a cell line exhibiting epithelial morphology that was isolated from a solid, invasive ductal carcinoma of the breast obtained from a 60‐year‐old, female cancer patient. Cancer cells plated on glass coverslips in DMEM (Biological Industries, Kibbutz Beit‐Haemek, Israel) with 10%‐FBS (Biological Industries, Kibbutz Beit‐Haemek, Israel) and 1% GlutaMAX (Gibco, New York, USA) culture medium. The cultures were grown in the incubator for 40%–50% confluence under standard conditions (37°C and 95% humidity).

### 2.4 Live Cell Imaging

Fluorescence measurements were performed using a Zeiss LSM 900 (Carl Zeiss Microscopy GmbH, Jena, Germany) and a 20x or 63x oil immersion objective. Illumination intensity was kept to a minimum (0.1%–0.2% of laser output) to avoid phototoxicity.

### 2.5 mPTP Opening Visualization

**Figure 1:**
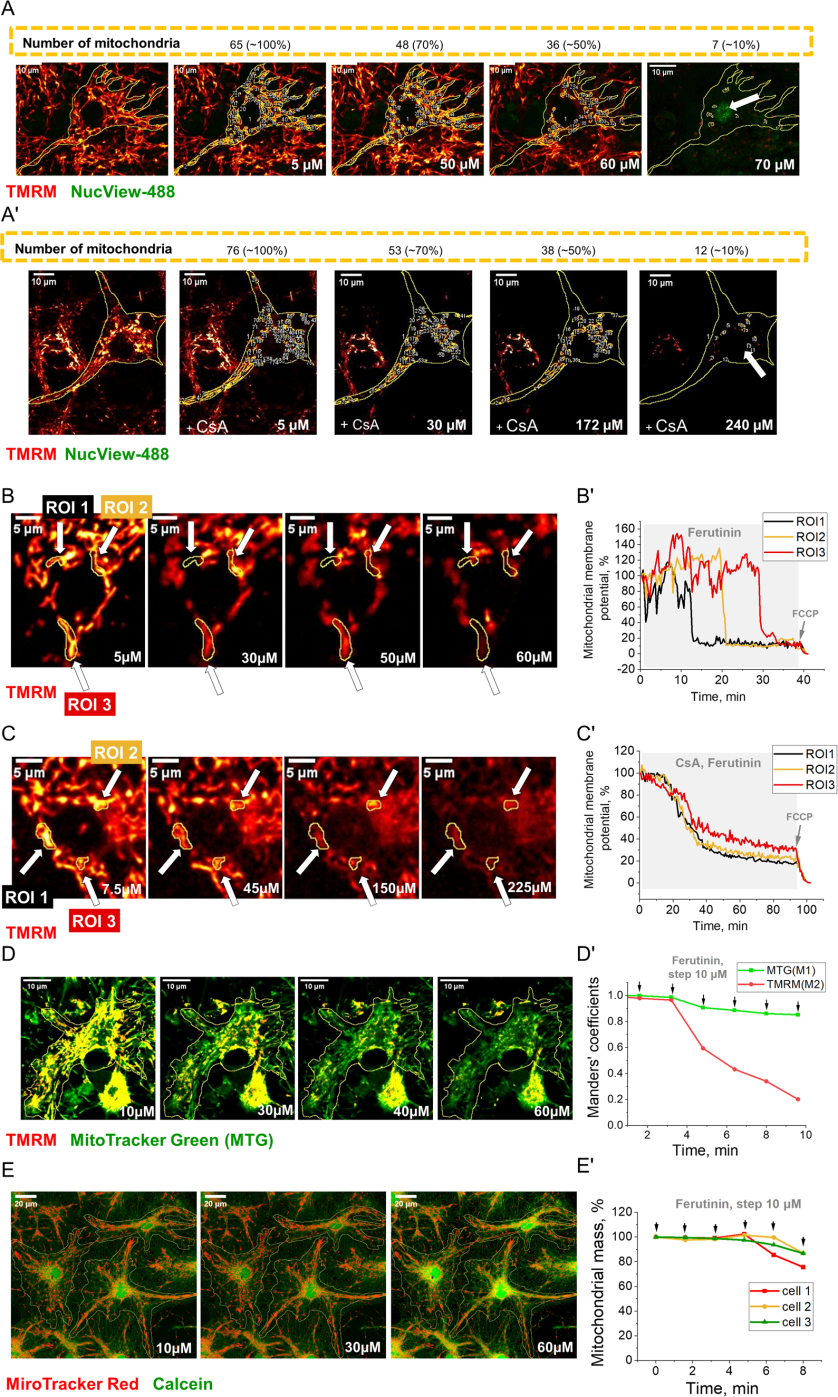
Measurement of cyclosporine‐sensitive mitochondrial permeability transition pore (mPTP) opening. Neurons in control neuron‐glial culture (A) and neurons in neuron‐glial culture with preincubation of 0.5 μM of cyclosporine A (A'). The numbers indicate different concentrations of ferutinin during stepwise addition (corresponding concentration of ferutnin μM). The cells were loaded with tetramethylrhodamine methyl ester (TMRM) probes (red color) and NucView‐488 probes (green color). Region of interest (ROIs) show counted mitochondria. The numbers above indicate the percentage of individual mitochondria remaining in the cells when the appropriate concentration of ferutinin was added compared to when the ferutinin concentration was 0. Images of neuronal mitochondria loaded with TMRM, demonstrating the fast loss of mitochondrial potential under the influence of stepwise ferutinin addition (μM) (B) and the delayed progressive loss of mitochondrial potential (C) under the combined action of preincubation with of 0.5 μM of cyclosporine A and the action of ferutinin. The numbers indicate different concentrations of ferutinin during stepwise addition (corresponding concentration of ferutnin μM). ROIs indicates selected mitochondria. Traces shows the change in mitochondrial membrane potential (%) of selected ROIs over time upon addition of ferutinin (B') alone and ferutinin after preincubation with 0.5 μM of cyclosporine A (C'). Simultaneous monitoring of mitochondrial mass and membrane potential during stepwise ferutinin treatment in neuron‐glial culture. Representative confocal image of neuron‐glial culture co‐stained with MitoTracker Green (MTG, green) and TMRM (red). Yellow color indicates colocalization of both signals. Scale bar 10 μm (D). Quantitative analysis of mitochondrial signals during stepwise ferutinin addition (10 μM indicated by arrows). Green line shows Manders' coefficient M1 representing MTG signal (mitochondrial mass), which remains relatively stable (~85% of initial value at 60 μM ferutinin). Red line shows Manders' coefficient M2 representing TMRM signal (polarized mitochondria), which progressively decreases to ~20% of initial value at 60 μM ferutinin. The divergence between these signals demonstrates that mitochondrial structures remain largely intact despite progressive loss of membrane potential (D'). Analysis of mitochondrial mass preservation during ferutinin treatment in neuron‐glial cultures using Calcein and MitoTracker Red co‐staining. Representative confocal image of neuron‐glial culture co‐stained with Calcein (green, showing cell bodies and processes) and MitoTracker Red (red, labeling mitochondria. Scale bar 20 μm (E). (B) Quantitative analysis of mitochondrial mass in three individual cells during stepwise ferutinin treatment (10 μM indicated by arrows). The mitochondrial mass remains largely preserved (80%–100% of initial value) even at high ferutinin concentrations, with only minor variations between individual cells. Small decrease in mitochondrial mass observed in cell 1 at higher ferutinin concentrations may reflect partial dependence of MitoTracker Red accumulation on membrane potential. Data represent mitochondrial mass calculated as percentage of initial values for each cell (E').

It is assumed that the sharp loss of mitochondrial membrane potential (ΔΨm) is due to the of mPTP opening. Cells (neuron‐glial culture, fibroblasts, cancer cells) were loaded with mitochondrial potential‐sensitive 25 nM tetramethylrhodamine methyl ester (TMRM) (Invitrogen by Thermo Fisher Scientific, USA) and 2 μM fluorescent caspase‐3 substrate NucView‐488 (Invitrogen by Thermo Fisher Scientific, USA) in a HEPES‐buffered HBSS for 45 min at a room temperature and during visualization TMRM and NucView‐488 remained in the HBSS. Next, a stepwise addition of electrogenic calcium ionophore ferutinin (Abcam, USA) which overload mitochondria by Ca^2+^ and can act as an inducer of mPTP opening (Abramov 2003; Zamaraeva et al. 1997) until the fluorescence of NucView‐488 began (apoptosis induction). After each ferutinin addition, the number of mitochondria was manually counted by TMRM fluorescence as shown in the Figure 1А, as well as the mitochondrial area was calculated using “Particle analyzer” Fiji (Open‐source software) plugin.

To confirm the mPTP‐dependent mitochondria depolarization, an experiment was carried out with the ferutinin addition and preincubation of cells with 0.5 μM Cycloporin A (Sigma Aldrich, USA) for 30 min. Cyclosporin A is an inhibitor of the mPTP. As can be seen from Figure 1B, in the presence of Cyclosporine A mitochondrial depolarization occurs at higher concentrations of ferutinin compared to untreated cells, but even at high concentrations (more than 200 μM) the caspase‐3 activation does not occur. Figure 1C,D shows the difference between the fast and transient loss of mitochondrial potential under the influence of ferutinin and the slow progressive loss under the combined action of cyclosporine and ferutinin. Cyclosporine A inhibits transient loss of potential, but not slow progressive loss.

### 2.6 Cell Death

For assessment of cell death cells were incubated with 5 μM Hoechst 33342 (Invitrogen, Oregon, USA) and 20 μM Propidium iodide (PI, Invitrogen, USA) and NucView‐488 (Invitrogen by Thermo Fisher Scientific, USA) in a HEPES‐buffered HBSS for 45 min at RT. Hoechst stains chromatin DNA in all cells, PI iodide can only stain permeated to be dead cells and NucView‐488 detects cells with activated caspase‐3. We measured cell death: necrotic (PI‐positive) relative to total number of cells (Hoechst) in control. Cell density was also taken into account when calculating cell death percentage.

### 2.7 mRNA Measurement

The work was carried out on primary rat neuron‐glial culture, human fibroblasts, BT‐474 cancer cell line (origin—human). Total RNA (to tRNA) was isolated from the cell culture utilizing the RNA Solo kit (Eurogen), following the manufacturer's instructions. The quantity of isolated totRNA was determined spectrophotometrically at 260 nm (NanoDrop). cDNA synthesis was undertaken employing MMLV RT (Eurogen). The reverse transcription reaction solution consisted of the subsequent (total volume: 20 μL): 1–2 μg to tRNA, 20 μM oligo (dT) primers, 10 mM dNTP, 20 mM DTT, and 100 units of reverse transcriptase MMLV RT in reverse transcription buffer. The reaction proceeded for 45 min at 40°C; it was terminated by heating the mixture for 10 min at 70°C. The resulting cDNA preparations were used as a template for real‐time PCR. The PCR reaction mixture was composed of: 3 μL of a cDNA specimen, a blend of reverse and forward primers (10 μM each), and a qPCRmix‐HS SYBR reaction mixture (Eurogen) enclosing Taq DNA polymerase. Following initial denaturation (5 min at 95°C), 40 amplification cycles were conducted: denaturation at 95°C for 30 s, annealing at a temperature particular to the primer for 20 s, and elongation at 72°C for 30 s. Expression of the studied genes was evaluated by real‐time PCR operating the DTlite amplifier (NPO DNA‐Technology LLC, Russia). To determine totRNA contamination with impurities of genomic DNA, a negative control reaction was established. In these specimens, the fluorescent signal was absent, denoting the absence of genomic DNA impurities. The relative expression level was calculated using the $2^{–\Delta {С}_{t}}$ method, where the GAPDH gene was taken as a reference gene (glyceraldehyde‐3‐phosphate dehydrogenase) (Kozera and Rapacz 2013). The oligonucleotides sequence used in the work (Eurogen, Russia) is shown below (Table 1).

**Table 1:** The oligonucleotides sequence used in the measurements.

Primer name	Primer	Sequence (human)	Sequence (rat)
Gapdh	F	TGGTCGTATTGGGCGCCTGG	CCACGGCAAGTTCAACGGCAC
	R	GAGGGGGCAGAGATGATGACCCTTTT	GATGATGACCCTTTTGGCCCCACC
Bcl‐2	F	CTGCATCTCATGCCAAGGGGGA	TGGAGATGAAGACTCCGCGCCCCTGA
	R	CGTAGCCCCTCTGCGACAGCTTAT	CGTGGCAAAGCGTCCCCTCGCGGT
Bax	F	CGGCGGTGATGGACGGGT	GACACCTGAGCTGACCTTGGAGC
	R	GTCTGTGTCCACGGCGGCAAT	CGGGCACTTTAGTGCACAGGG
Bcl‐2l	F	GCCACTTACCTGAATGACCACC	GGGGCTATCCGCAGGTGC
	R	AACCAGCGGTTGAAGCGTTCCT	GAGCAGACCCAGTGATGAGCAGGT
Bak‐1	F	TTACCGCCATCAGCAGGAACAG	CCTGCTAACCCTGAGATGGAC
	R	GGAACTCTGAGTCATAGCGTCG	CGCTGGTAGACATACACCCC
ripk1	F	TATCCCAGTGCCTGAGACCAAC	GTGGTGAAGCTACTGGGCAT
	R	GTAGGCTCCAATCTGAATGCCAG	AGGAAGCCACACCAAGATCG
ripk3	F	GCTACGATGTGGCGGTCAAGAT	GCCACACAGCATGGAACCTT
	R	TTGGTCCCAGTTCACCTTCTCG	CGTACACGCGTAGTCCCACTCG
p53	F	CGACGGTGACACGCTTCCCTG	GCAGAGTTGTTAGAAGGCCCAGAG G
	R	GAAACCGTAGCTGCCCTGGTCGGT	GCCGTCACCATCAGCAACG
caspase 3	F	GGAAGCGAATCAATGGACTCTGG	CTGGACAGCAGTTACAAAATGGA
	R	GCATCGACATCTGTACCAGACC	TGACTTCGTATTTCAGGGCCA
caspase 8	F	GGACAGTGAAGATCTGGCCTCCCTC	CAAAGCCCAGGTTTCTGCCTA
	R	CACCCACCTGTAGCAGAAATTTGAGC	TCTCTGTGCTTGATCGTTCGT
caspase 9	F	GTTTGAGGACCTTCGACCAGCT	CTCTGCTGAGTCGTGAGCTCTTC
	R	CAACGTACCAGGAGCCACTCTT	CCAGGTGGTCTAGGGGTGTAA
parp1	F	CCAAGCCAGTTCAGGACCTCAT	TGGAATGAAGAAGCCCCCAC
	R	GGATCTGCCTTTTGCTCAGCTTC	TCATAGGCATTGTGCGTGGT

### 2.8 Statistical Analysis

OriginPro 2019 (Microcal Software Inc., Northampton, MA) was used for graphs generation and statistical analysis. Data are presented as mean and standard deviation (mean ± SD). Before hypothesis testing, all data were assessed for normality using the Shapiro–Wilk test. Parametric tests were used for all statistical comparisons due to all measured variables were found to meet normality assumptions (*p* > 0.05). Statistical significance was determined using one‐way ANOVA with Tukey's multiple comparison test, unless otherwise stated. The Bonferroni correction was used to adjust the significance level for multiple comparisons.

## 3 Results

### 3.1 Ferutinin Induce Dose‐Dependent mPTP Opening in Neurons and Caspase 3 Activation

**Figure 2:**
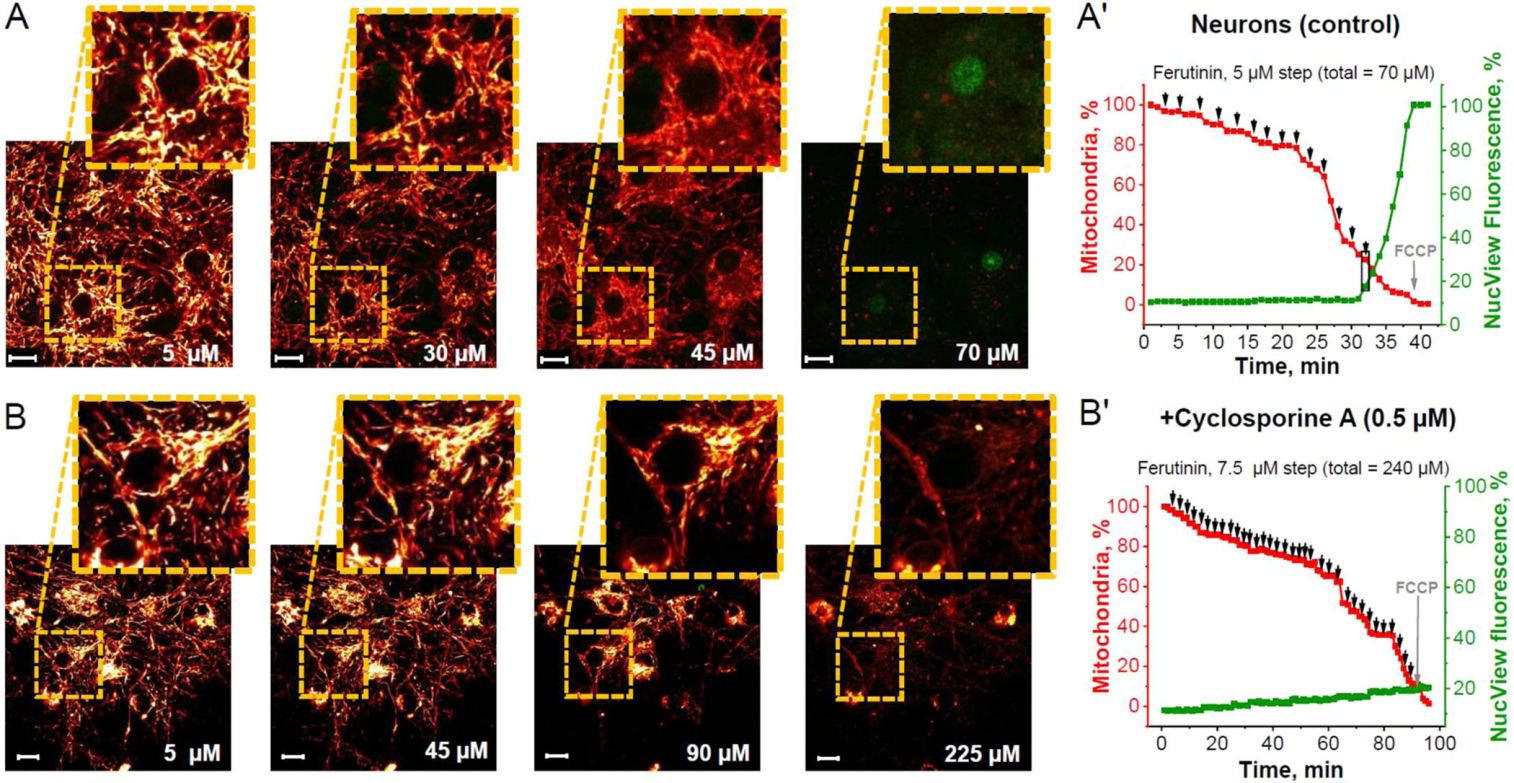
Inductions of mitochondrial permeability transition pore (mPTP)‐dependent apoptosis in neurons of neuron‐glial culture. Representative time‐course images demonstrating that caspase‐3 activation (green fluorescence of NucView‐488) is induced by ferutinin‐mediated mitochondrial permeability transition pore (mPTP) opening and loss of a critical number of neuronal mitochondria (red fluorescence of TMRM) in a neuron of control neuron‐glial cultures (A) and after preincubation with 0.5 μM cyclosporine A (B). Numbers indicate ferutinin concentrations in μM. Representative time traces showing stepwise ferutinin addition where percent of neuronal mitochondria compared to initial is in red and percent NucView‐488 fluorescence compared to maximal fluorescence after control caspase‐3 activation is in green: control neurons in neuron‐glial cultures (A') and after preincubation with 0.5 μM Cyclosporine A (B'). Black arrows on graph indicate ferutinin addition and black rectangle indicates moment of caspase‐3 activation for selected cell. Scale bar 10 μm.

Ferutinin induced two types of decrease of TMRM fluorescence—small and slow (which could be explained by the uncoupling action of ionophore) and fast and transient with complete loss of TMRM fluorescence in single mitochondria (Figure 1C,D). We took the second type of TMRM signal loss in single mitochondria as a unit of mPTP opening in experiments with ferutinin (Figures 1 and 2).

Dependence of the changes in TMRM signal on mPTP opening was also controlled with simultaneous MitoTracker Green (MTG)/TMRM imaging during stepwise ferutinin addition with quantitative colocalization analysis. As shown in the Figure 1D, we used Manders' colocalization coefficients (M1 and M2) as they specifically measure the fraction of overlapping signals independently of signal intensity variations: M1 represents the fraction of MTG signal coincident with TMRM signal, M2 represents the fraction of TMRM signal coincident with MTG signal (polarized mitochondria). Our analysis shows that while M2 (TMRM) progressively decreases with ferutinin treatment (to ~20% at 60 μM) while M1 (MTG) remains high (~85% of initial signal), indicating preservation of mitochondrial structures despite membrane potential loss. These results provide strong evidence that: (a) The rapid TMRM signal loss represents genuine depolarization events (b) Mitochondrial structures remain largely intact during the initial phases of ferutinin treatment, as demonstrated by both stable MTG signal and Manders' coefficient analysis c) The thresholds we describe in our study reflect true differences in sensitivity to mPTP opening rather than variations in mitochondrial degradation rates.

In contrast, the MTG signal remained relatively stable through the initial phases of TMRM loss, indicating preservation of mitochondrial mass. While we did observe some gradual decrease in MTG fluorescence, this can be attributed to the known partial potential‐dependence of MTG accumulation in mitochondria, rather than mitochondrial degradation.

The dynamics of these two signals—rapid TMRM loss versus relatively stable MTG—supports our interpretation that the primary event we observe is membrane potential dissipation rather than immediate mitochondrial degradation.

To understand how mitochondrial mPTP change the mitochondrial mass in cells we used simultaneous MitoTracker Red (MTR)/Calcein‐AM imaging during stepwise ferutinin addition. Quantitative analysis shows (Figure 1E) preservation of mitochondrial mass (80%–100%) and maintained cellular architecture throughout the experiment even at high ferutinin concentrations (60 μM).

The stepwise application of electrogenic calcium ionophore ferutinin to co‐culture of neurons and astrocytes induced a gradual decrease in mitochondrial number with TMRM fluorescence per neuron (Figure 2A,A'). At the total 70 μM (77.5 ± 9.8 μM) ferutinin adding, nucleolar fluorescence of NucView‐488 was increased, indicating activation of caspase‐3, corresponding to loss of 83 ± 8.6% of initial mitochondria or 70.5 ± 10.2% mitochondrial area in the cell (Figure 2A'). Importantly, incubation of the co‐culture of neurons and astrocytes with 0.5 μM cyclosporine A (Figure 2B) blocked the appearance of NucView‐488 fluorescence in nuclei, indicating that activation of caspase‐3 in neurons was not induced even with 240 μM (240 ± 13.6 μM) ferutinin. It should be noted that preincubation of the neurons with cyclosporine A inhibited fast and transient complete loss of TMRM fluorescence but not slow and progressive that brought to almost complete (> 95%) loss of TMRM signal in mitochondria (Figure 2B').

### 3.2 Astrocytes in Co‐Culture Have Different to Neurons Threshold of Mitochondria With mPT‐Induced Cell Death

**Figure 3:**
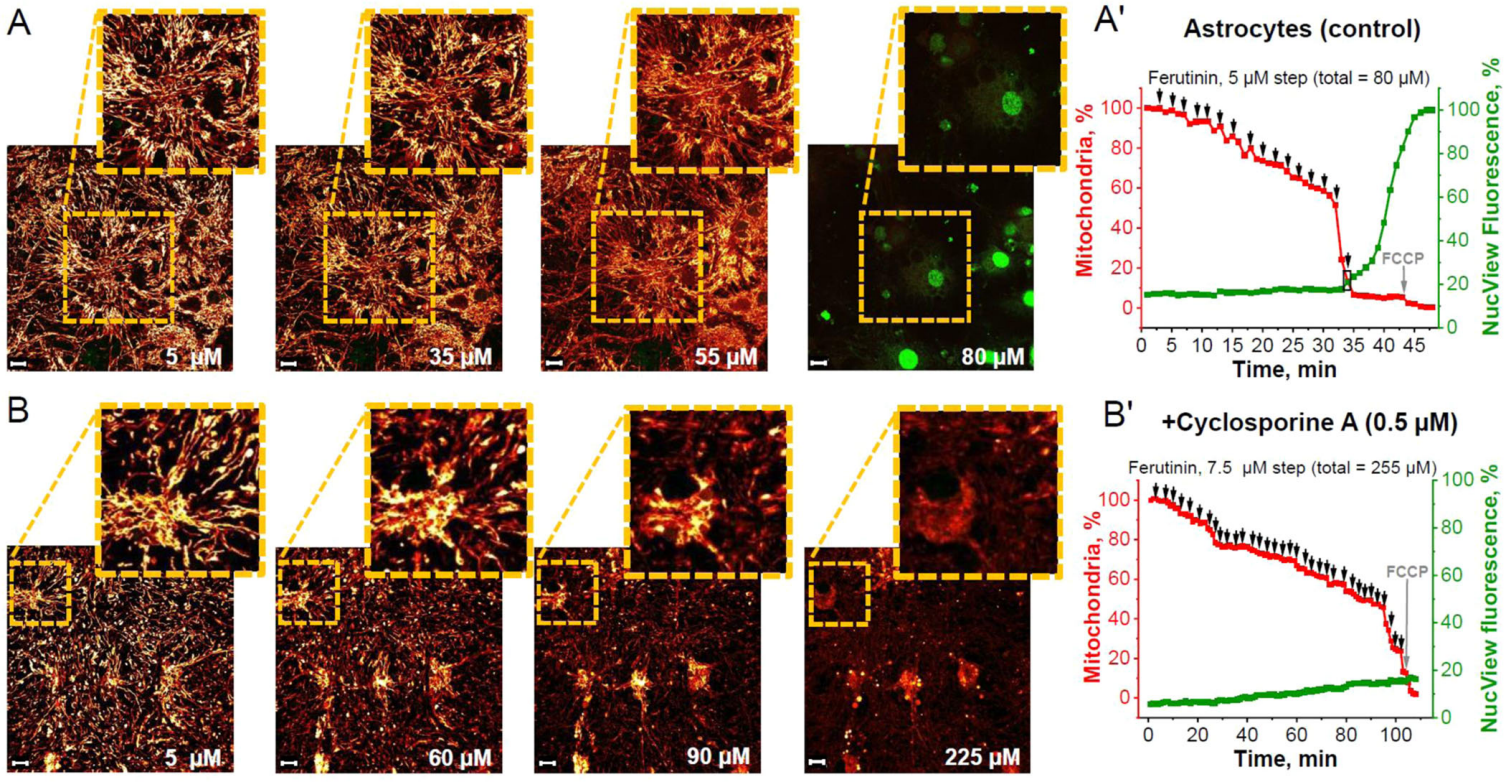
Inductions of mitochondrial permeability transition pore (mPTP)‐dependent apoptosis in astrocytes of neuron‐glial culture. Representative time‐course images that caspase‐3 activation (green fluorescence of NucView‐488) is induced by ferutinin‐mediated mitochondrial permeability transition pore (mPTP) opening and loss of a critical number of astrocyte mitochondria (red fluorescence of TMRM) in astrocyte of control neuron‐glial cultures (A) and after preincubation with 0.5 μM Cyclosporine A (B). Numbers indicate ferutinin concentrations in μM. Representative time traces showing stepwise ferutinin addition where percent of astrocyte mitochondria compared to initial is in red and percent NucView‐488 fluorescence compared to maximal fluorescence after control caspase‐3 activation is in green: control astrocytes in neuroglial cultures (A') and after preincubation with 0.5 μM Cyclosporine A (B'). Black arrows on graph indicate ferutinin addition and black rectangle indicates moment of caspase‐3 activation for selected cell. Scale bar 10 μm.

In astrocytes from the neuron‐glia co‐culture (Figure 3A), caspase‐3 activation occurred at 80 μM (87.5 ± 9.7 μM) of ferutinin, which corresponds to the loss of 90.2 ± 5.8% of initial mitochondria and 77.1 ± 5.9% loss of TMRM area in the cells (Figure 3A'). Similar to neurons, preincubation of astrocytes with 0.5 μM cyclosporine A (Figure 3B) blocked the appearance of NucView‐488 fluorescence (activation of caspase‐3) even with 255 μM ferutinin (249.3 ± 12.3 μM). Loss of mitochondria in these experiments was overall slower than without cyclosporine A (Figure 3B').

### 3.3 Fibroblasts Have Lower mPTP Threshold to Induction of Apoptosis

**Figure 4:**
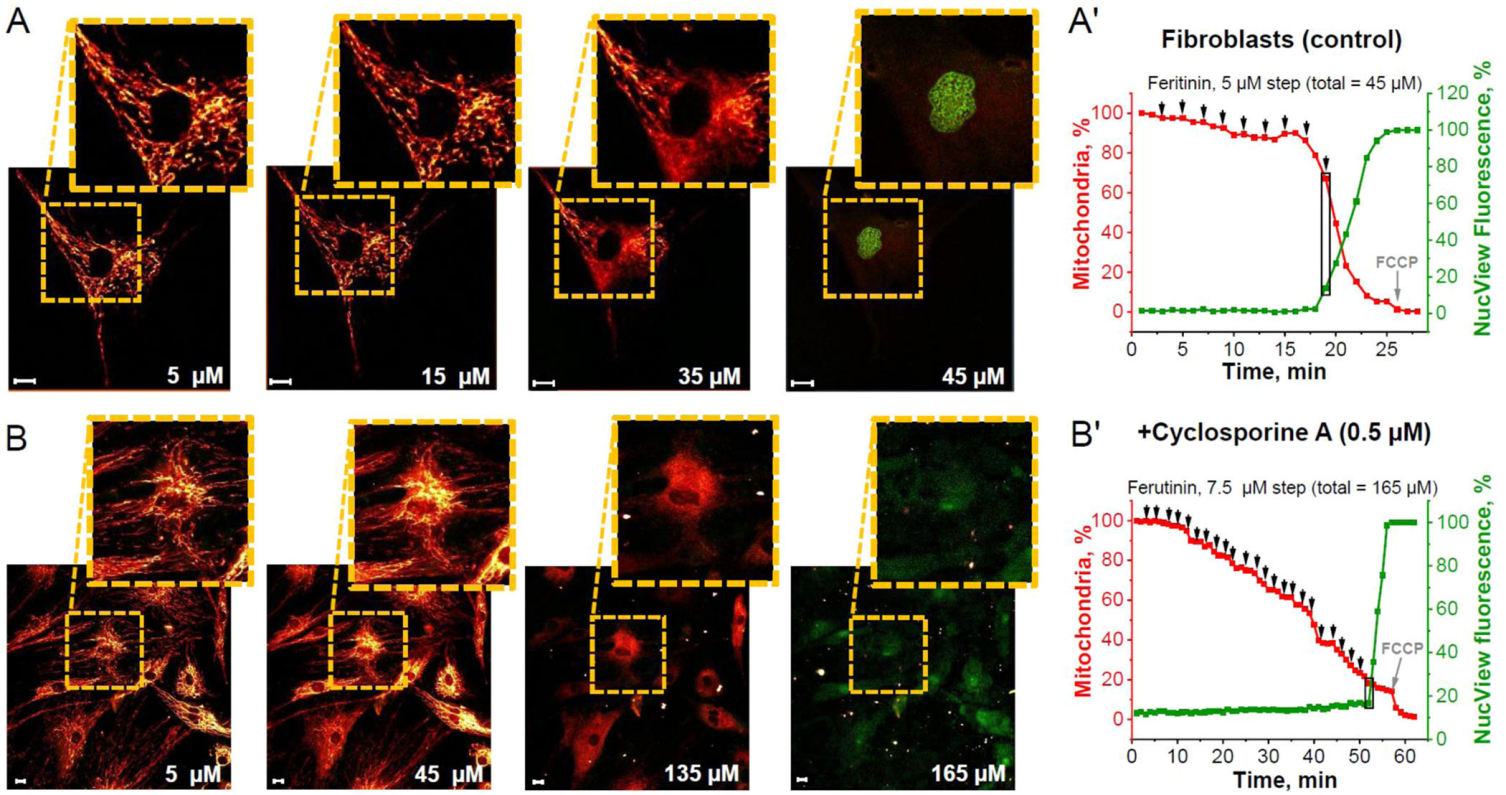
Inductions of mitochondrial permeability transition pore (mPTP)‐dependent apoptosis in skin fibroblasts. Representative time‐course images demonstrating that caspase‐3 activation (green fluorescence of NucView‐488) is induced by ferutinin‐mediated mitochondrial permeability transition pore (mPTP) opening and loss of a critical number of fibroblast mitochondria (red fluorescence of TMRM) in control fibroblast culture (A) and after preincubation with 0.5 μM Cyclosporine A (B). Numbers indicate ferutinin concentrations in μM. Representative time traces showing stepwise ferutinin addition where percent of fibroblast mitochondria compared to initial is in red and percent NucView‐488 fluorescence compared to maximal fluorescence after control caspase‐3 activation is in green: control fibroblasts in cultures (A') and after preincubation with 0.5 μM Cyclosporine A (B'). Black arrows on graph indicate ferutinin addition and black rectangle indicates moment of caspase‐3 activation for selected cell. Scale bar 10 μm.

Fibroblasts, in contrast to neurons, are cells which are constantly renewing and they could have different vulnerability to mPTP opening. We also investigated the mitochondrial loss (mitochondria with open mPTP) threshold for apoptosis activation in normal skin fibroblasts (Figure 4A). Caspase‐3 in fibroblasts was activated by the loss of 35.1 ± 7.8% of initial mitochondria and 26 ± 4.4% loss of mitochondrial area at 45 μM ferutinin (33.2 ± 8.1 μM) (Figure 4A'). In contrast to neurons and astrocytes, fibroblasts preincubated with 0.5 μM cyclosporine A (Figure 4B) enhanced mitochondrial integrity and delayed, but did not prevent, caspase‐3 activation. In this case, caspase‐3 activation occurred at 165 μM ferutinin (150 ± 23.7 μM) and required loss of more initial mitochondria (83.2 ± 9.5%) and mitochondrial area (74.4 ± 7.1%) compared to untreated fibroblasts (Figure 4B').

### 3.4 mPTP Opening and Apoptosis in Cancer BT‐474 Cells

**Figure 5:**
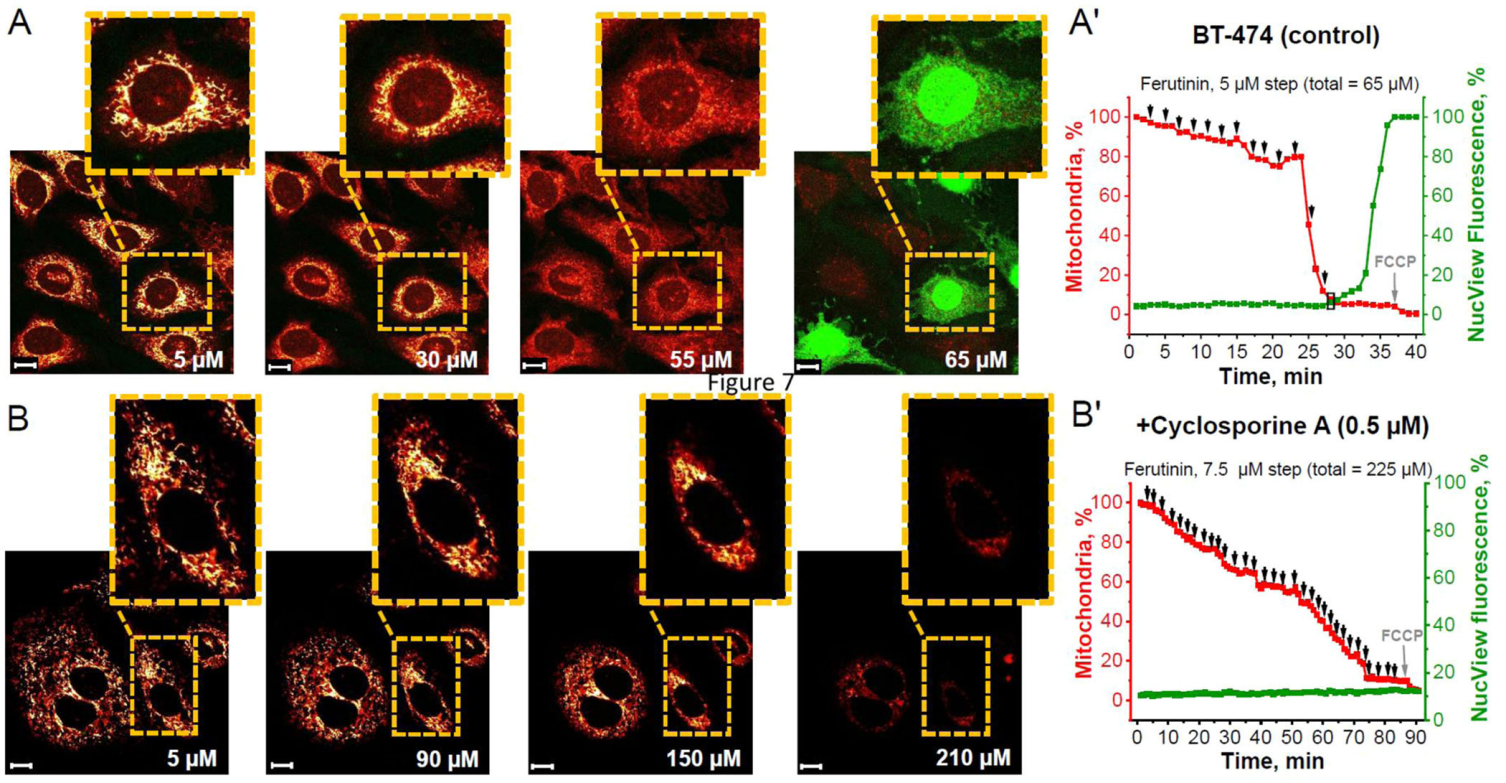
Inductions of mitochondrial permeability transition pore (mPTP)‐dependent apoptosis in BT‐474 cancer cells. Representative time course images showing that that caspase‐3 activation (green fluorescence of NucView‐488) is induced by ferutinin‐mediated mitochondrial permeability transition pore (mPTP) opening and loss of a critical number of BT‐474 cancer cell mitochondria (red fluorescence of TMRM) in control culture (A) and after preincubation with 0.5 μM of Cyclosporine A (B). The numbers indicate different ferutinin concentrations in μM. Representative time traces showing stepwise ferutinin addition where the percent of BT‐474 cancer cells mitochondria relative to the initial moment is in red and the percent of NucView‐488 fluorescence relative to the maximum fluorescence after control caspase‐3 activation is in green: control BT‐474 cancer cells culture (A') and after preincubation with 0.5 μM of Cyclosporine A (B'). Black arrows on the graph indicate the addition of ferutinin and the black rectangle indicates the moment of caspase‐3 activation for the selected cell. Scale bar 10 μm.

We next examined how many mitochondria per cell should open mPTP to induce apoptosis in cancer cells (Figure 5A). Ferutinin was gradually applied to BT‐474 cancer cells and caspase‐3 activation was observed at a concentration of 65 μM ferutinin (60 ± 7.6 μM), which corresponded to the loss of 90.4 ± 6.1% of initial mitochondria and 90.6 ± 7.6% of mitochondrial area (Figure 5A').

Preincubation of BT‐474 cancer cells with 0.5 μM Сyclosporine A prevented caspase‐3 activation in response to ferutinin (Figure 5B). Thus, complete loss of mitochondrial potential in BT‐474 cancer cells with CsA treatment also occurred at higher concentrations of ferutinin (208.2 ± 19.7 μM) compared to untreated cells (Figure 5B').

*The threshold of mitochondria with mPTP for induction of apoptosis varies between cell types.*

**Figure 6:**
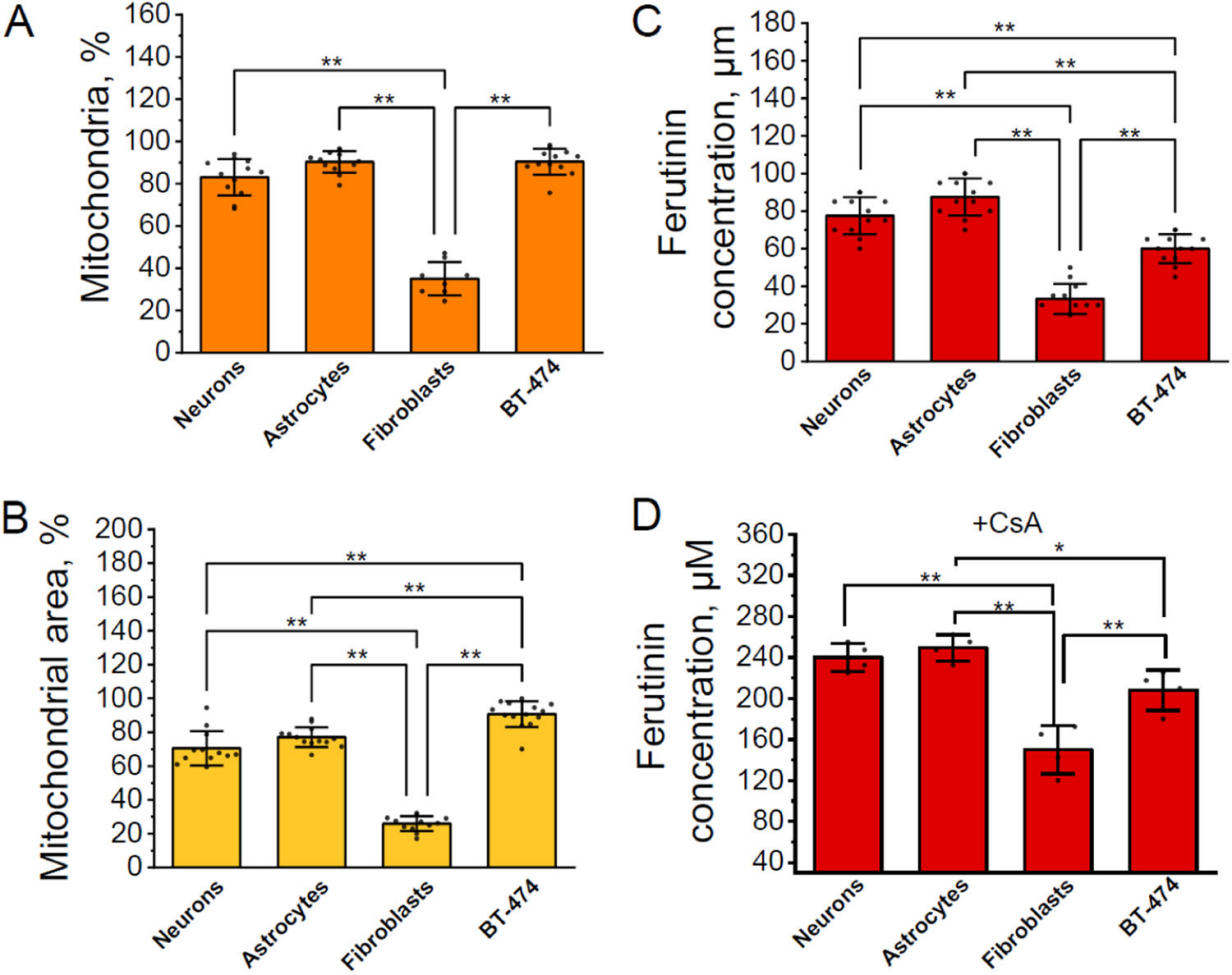
Relationship between mitochondrial permeability transition pore (mPTP) opening and apoptosis induction in different cell types. Graph showing the percentage of mitochondria (A) and mitochondrial area (B) from the initial measured by tetramethylrhodamine methyl ester (TMRM) fluorescence required to activate caspase‐3 indicated by NucView‐488 fluorescence in neurons and astrocytes of neuron‐glial culture, skin fibroblasts, BT‐474 cancer cells. Ferutinin concertation (μM) required for caspase‐3 activation indicated by NucView‐488 in neurons and astrocytes of neuron‐glial culture, skin fibroblasts, BT‐474 cancer cells (C). Ferutinin concertation (μM) required for loss > 95% of initial mitochondria measured by tetramethylrhodamine methyl ester (TMRM) fluorescence after preincubation with 0.5 μM of Cyclosporine A in neurons and astrocytes of neuron‐glial culture, skin fibroblasts, BT‐474 cancer cells (D). *n* = 12 cells in four independent experiments. Bar plots show mean ± SD. Dots represent individual data points. Statistical significance was determined using one‐way ANOVA with Tukey's multiple comparison test. The Bonferroni correction was used to adjust the significance level for multiple comparisons (adjusted *p* = 0.005; *p < 0.005, ** *p* < 0.001).

Using the data from the above‐described experiments we have studied the differences in the number of mitochondria with mPTP which induce apoptosis in different cells (Figure 6). However, the shape of single mitochondria and their volume may be different, therefore we measured another parameter—mitochondrial area remaining from baseline as measured by TMRM fluorescence when caspase‐3 activation (NucView488 fluorescence) was activated. Total concentration of ferutinin which induces apoptosis may also reflect calcium sensitivity of the cells to mPTP and apoptosis. Neurons and astrocytes from a co‐culture show no difference for triggering apoptosis with caspase‐3 activation induced by ~80% loss of initial mitochondria signal (Figure 6A) and ~90% loss of mitochondrial area (Figure 6B), corresponding to ~80 μM ferutinin (Figure 6C).

In contrast, skin fibroblasts activate caspase‐3 with only ~35% of mitochondria with mPTP opening that also reflects smaller mitochondrial area (Figure 6A,B). Importantly, ferutinin induces apoptosis in fibroblasts at threefold lower concentration compared to neurons and astrocytes (Figure 6C). Compared to fibroblasts, the transformed BT‐474 cancer line demonstrated caspase‐3 activation at nearly 2‐fold higher ferutinin levels (Figure 6С) but with over 2.5‐fold higher mitochondrial loss (Figure 6A,B).

Preincubation with the mPTP inhibitor, cyclosporin A, revealed differences in sensitivity to mitochondrial damage in neuron‐glial cultures, fibroblasts and cancer cell line compared to untreated cultures (Figure 6D). Interestingly, caspase‐3 activation was not observed in both neurons and astrocytes from neuron‐glial culture, even with a loss of mitochondria of more than 95% and a threefold higher concentration of ferutinin (Figure 6D). In fibroblasts, upon preincubation with cyclosporine A, the threshold for caspase‐3 activation, which depends on mitochondrial damage, was significantly increased by 2.6 times, and it also required a threefold higher concentration of ferutinin compared to untreated fibroblasts. However, BT‐474 cancer cells with cyclosporine A did not undergo apoptosis even at > 95% mitochondrial loss and 3.5‐fold higher ferutinin concentrations compared to untreated cells (Figure 6D). Thus, cyclosporine A increased the resistance of all studied cell types to the action of the calcium ionophore—ferutinin, revealing the mitochondrial integrity in general and mPTP in particular, as central players in the regulation of apoptosis.

mPTP‐induced necrosis in neurons, astrocytes, fibroblasts, and BT‐474 cancer cells.

**Figure 7:**
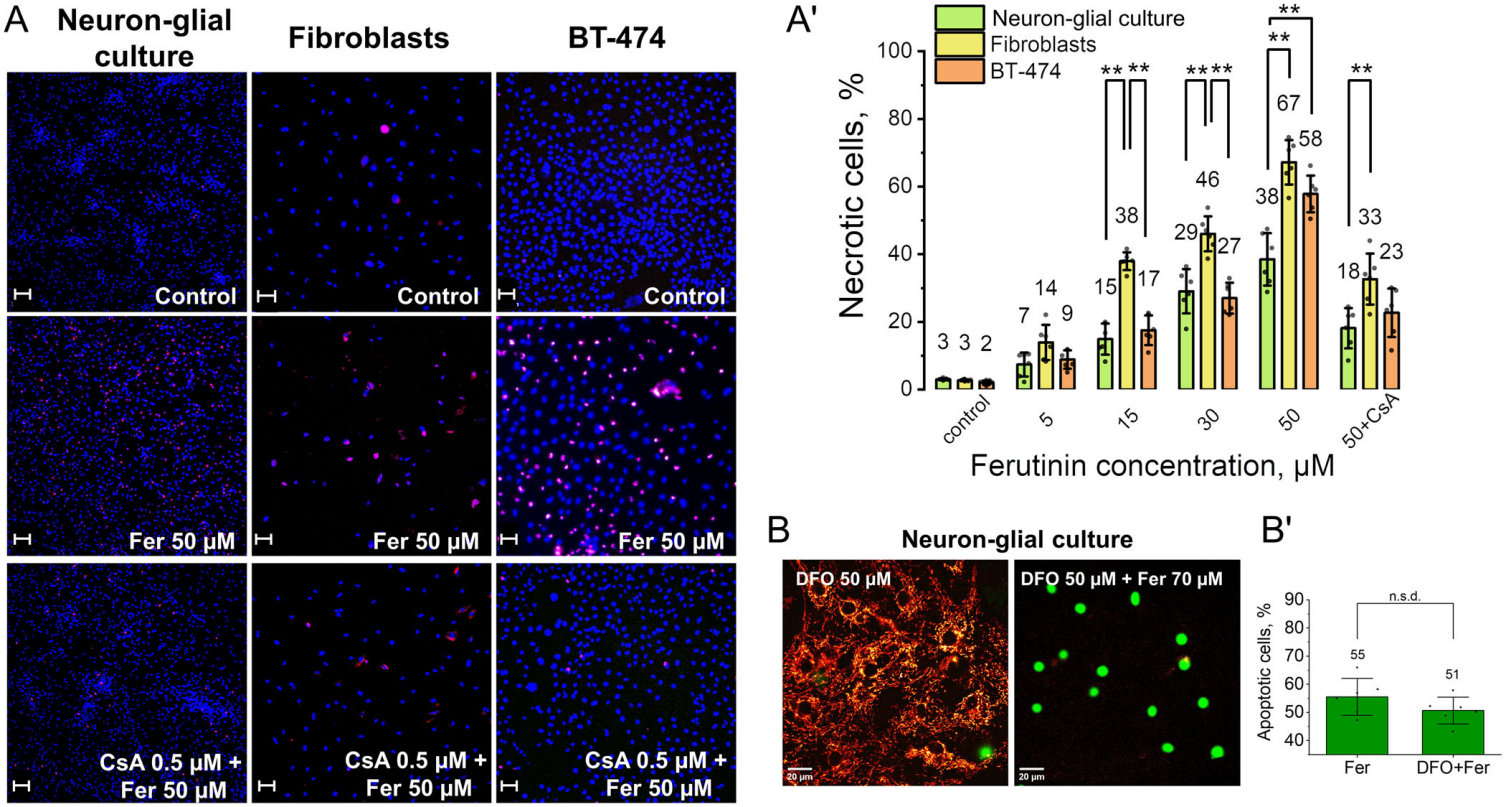
Effect of ferutinin on the necrosis in neuron‐glial culture, fibroblasts and BT‐474 cancer cells. Images of rat primary neuron‐glial culture, fibroblasts culture, and BT‐474 cancer cells culture labeled with Hoechst 33342 (blue pseudocolor), PI (red pseudocolor): control, after ferutinin addition (50 μM for 24 h) and after preincubation with Cyclosporine A (0.5 μM) and ferutinin addition (50 μM for 24 h). Scale bar 50 μm (A). Percentage of necrotic cells (Hoechst 33342 and PI‐positive), relatively total number of cells in untreated control after 24‐h incubation with different concentrations of ferutinin in neuron‐glial culture, BT‐474 cancer cells and fibroblasts and percentage of dead cells after preincubation with 0.5 μM of Cyclosporine A (CsA) and 24‐h incubation with 50 μM of ferutinin. *n* = 6 independent experiments. Bar plots show mean ± SD. Statistical significance between cell types was determined using one‐way ANOVA with Tukey's multiple comparison test. The Bonferroni correction was used to adjust the significance level for multiple comparisons (adjusted *p* = 0.05; ** *p* < 0.001) Statistical significance of the necrotic cells level with CsA compared to the necrotic cells with 50 μM of ferutinin was determined using an unpaired Student's *t*‐test (** *p* < 0.001) (A'). Representative confocal image of rat primary neuron‐glial culture pretreated with the ferroptosis inhibitor deferoxamine (DFO, 50 μM, 1 h) and co‐stained with TMRM (red, indicating mitochondrial membrane potential) and NucView‐488 (green, indicating caspase‐3 activation) before and after stepwise ferutinin treatment (total concentration 70 μM). Scale bar 20 μm (B). Percentage of apoptotic cells (NucView‐488‐positive) in neuron‐glial culture, relatively total number of cells after stepwise ferutinin addition (total concentration 70 μM) and pretreatment with the DFO (50 μM, 1 h) and ferutinin addition (B'). *n* = 3 independent experiments. Bar plots show mean ± SD.

To identify if necrosis resulting from ferutinin‐induced mitochondrial calcium overload is also sensitive to mPTP opening across cell types, we incubated neuron‐glial cultures, fibroblasts, and BT‐474 cancer cells for 24 h with ferutinin concentrations which induced mPTP in cells (5, 15, 30, and 50 μM). Cells were co‐stained with the live‐cell permeant dye Hoechst 33342 and Propidium Iodide (PI), which only enters cells with permeable plasma membranes indicating necrosis (Figure 7B). We observed dose‐dependent induction of necrosis by ferutinin in all cell types tested (Figure 7A). However, at 15, 30, and 50 μM ferutinin, fibroblasts displayed significantly higher percentages of PI‐positive necrotic cells (15 μM: 37.9 ± 2.6%; 30 μM: 46.2 ± 5.1%; 50 μM: 67.1 ± 6.5%) compared to neuron‐glial cultures (15 μM: 14.8 ± 4.6%; 30 μM: 29.0 ± 6.5%; 50 μM: 38.4 ± 7.7%) and BT‐474 cancer cells (15 μM: 17.4 ± 4.3%; 30 μM 27.0 ± 4.5%; 50 μM: 57.8 ± 5.4%). Pretreatment of cells with mPTP inhibitor cyclosporine A (0.5 μM) significantly reduced 50 μM ferutinin‐induced necrosis in all cell types compared to no pretreatment (neuron‐glial: 18.1 ± 5.9%; fibroblasts: 32.6 ± 7.5%; BT‐474: 22.6 ± 7.1%). Thus, besides the apoptotic effect of stepwise addition of ferutinin, the excess calcium load caused by an acute high dose of ferutinin leads to necrosis, especially pronounced in fibroblasts. Mitochondrial membrane permeability also plays an important role in mediating this damage, as confirmed by experiments with cyclosporine A.

Although ferroptosis is strongly associated with oxidative stress it also can be calcium sensitive (Angelova et al. 2020; Angelova and Abramov 2024) and potentially dependent on the calcium dependent mPTP opening. Pretreatment of cells with inhibitor of ferroptosis, iron chelator Deferoxamine (DFO) (50 μM, 1 h) did not prevent ferutinin‐induced cell death (Figure 7B,B'). The inability of DFO to prevent cell death, together with the clear mitochondrial and caspase‐dependent characteristics we observe, indicates that iron‐dependent cell death pathways are not significantly involved in ferutinin's mechanism of action.

The number of mitochondria with mPTP which triggers apoptosis does not correlate with the expression levels of proapoptotic or apoptotic proteins in these cells, but correlates with the expression ratio of pro‐ and antiapoptotic genes Bax/Bcl‐2.

Considering the difference in percentage of mitochondria with mPT which induces cell death in different cell types, there should be a mechanism of selective sensitivity to this trigger in different cells. One of the possible explanations could be the difference in the level of proapoptotic proteins in mitochondria which are released after mPTP opens or higher expression levels of apoptotic proteins (such as caspases 3–9).

**Figure 8:**
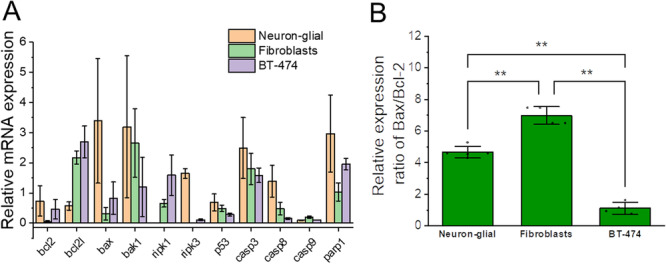
mRNA expression of protein, related with apoptosis, necrosis and cell reparation in neuron‐glial culture, fibroblasts, and B‐474 cancer cells examined by RT‐qPCR analysis. (A) Relative mRNA expression of pro‐ and antiapoptotic proteins – Bax, Bcl‐2, Bcl‐2l, Bak‐1, apoptosis/necrosis‐related proteins ripk1, ripk3, and p53 and caspases which related to apoptosis—3, 8, 9, and parp1 in neuron‐glial culture, fibroblasts, and BT‐474 cancer cells examined by RT‐qPCR analysis and calculated using the $2^{‐\Delta {С}_{t}}$ method, where GAPDH gene was taken as a reference. (B) Bax/Bcl2 relative mRNA expression ratio in neuron‐glial culture, fibroblasts and B‐474 cancer examined by RT‐qPCR analysis and calculated using the $2^{‐\Delta {С}_{t}}$ method, where GAPDH gene was taken as a reference. *n* = 4 independent experiments. Bar plots show mean ± SD. Dots represent individual data points. Statistical significance was determined using one‐way ANOVA with Tukey's multiple comparison test. The Bonferroni correction was used to adjust the significance level for multiple comparisons (adjusted *p* = 0.05; ** *p* < 0.001).

We examined the expression of pro‐ and antiapoptotic proteins—Bax, Bcl‐2, Bcl‐2l, Bak‐1, apoptosis/necrosis‐related proteins ripk1, ripk3, and p53 and caspases which related to apoptosis—3, 8, 9, and parp1 in neuron‐glial culture, fibroblasts and BT‐474 cancer cells by RT‐qPCR analysis. Expression of these proteins in cell cultures were calculated using the $2^{‐\Delta {С}_{t}}$ method, where the GAPDH gene was taken as a reference gene (Figure 8A). We have found no correlation between expression of these proteins and the vulnerability of cells to mPTP opening and apoptosis (Figure 8A). However, the ratio of the main pro‐ and antiapoptotic cells involved in programmed cell death—Bax/Bcl‐2 has shown a difference between cell types. Thus, BT‐474 cells displayed the lowest Bax/Bcl‐2 ratio (1.1 ± 0.3), which is significantly lower compared to the other cell types, while the highest significant Bax/Bcl‐2 expression ratio was in fibroblasts (6.9 ± 0.5). The Bax/Bcl‐2 expression ratio for neuron‐glial culture (4.6 ± 0.3) was significantly higher than this ratio for B‐474 cancer cells and less than the Bax/Bcl‐2 expression ratio for fibroblasts. The ranked the order of Bax/Bcl‐2 expression from highest to lowest and the outcome was fibroblasts > neuron‐glial cultures > BT‐474 cancer cells. This expression ratio of Bax/Bcl‐2 may explain the different sensitivity to the induction of apoptosis in calcium overload with ferutinin in the studied cell cultures.

## 4 Discussion

One of the major difficulties in the study of mPTP and the interpretation of results in whole and intact cells is the choice of methods which are used to assess this process. Here we use simultaneous measurement of Δψm (TMRM) and initiation of apoptosis (measures as a caspase‐3‐cleaved fluorescent substrate, NucView488). As a trigger for mPTP induction we used mitochondrial calcium overload which in intact cells could be induced by electrogenic calcium ionophores which is able to transport Ca^2+^ due to charge even against Ca^2+^ gradient in contrast to electroneutral ionomycin and A23187 (Abramov et al. 2001; Abramov 2003). The electrogenic calcium ionophore ferutinin is shown to not only be able to induce mPTP but also to induce apoptosis (Kovac et al. 2019; Macho et al. 2004). In our experiments ferutinin induced dose‐dependent transient loss of Δψm in single mitochondria that facilitated us to calculate the ratio between number of mitochondria with mPTP and induction of the caspase‐3 activation and initiation of cell death. Knowing the concentrations of ferutinin which induce mPTP we have managed to identify the proportion of mitochondria with mPTP which can be involved in necrosis.

Apoptosis, or programmed cell death, is a tightly‐regulated process critical for development and tissue homeostasis. Permeabilization of the mitochondrial membrane by opening of the mitochondrial permeability transition pore (mPTP) may be the “point of no return” in the initiation of programmed cell death during various pathologies, including neurodegeneration, cardiovascular diseases, diabetes, and so forth (Baev et al. 2024; Giorgi et al. 2012; Wang and Youle 2009). Considering this, an effective system which regulates the processes of apoptosis triggering should exist. Here we found that in cells, where activation of apoptosis is essential physiological step in the mechanism of the tissue regeneration—skin fibroblasts, activation of the caspase‐3 induced by much smaller percentage of mitochondria with opened mPTP compared to cells where apoptosis induces pure pathological death of neurons and astrocytes. However, not all cells undergo apoptosis with equal sensitivity (Hao and Mak 2010; Özören and El‐Deiry 2002) and dynamics of mPTP opening may play a key role in this specific sensitivity. This study shows differences between neural, dermal and cancerous cell types with regard to apoptosis induction thresholds following ferutinin‐mediated disruption of the mitochondrial network. While cortical neurons and astrocytes remained stable unto ~80%–90% loss of mitochondrial area (Figures 2 and 3), fibroblasts activated caspase‐3 only at ~35% mitochondrial damage (Figure 4). Remarkably, BT‐474 cancer cells underwent more than 90% mitochondrial loss before apoptotic commitment (Figure 5).

In our view, our results also answer the important question whether mPTP opening is purely an event leading to cell death or it also could be involved in physiological processes in the cells which has been suggested in the number of publications (Brenner and Moulin 2012; Carraro and Bernardi 2023). Here we show that mPTP in cells could be open without the induction of cell death but this only until reaching the cell‐specific threshold. Mitochondrial PTP flickering, which is mainly induced by various ROS production (Bhagatte Lodwick and Storey 2012; Blanchet et al. 2014; Novikova et al. 2022) also shown to be non‐pathological event probably also due to insufficient number of mitochondria with permeability transition.

The combination of MTG/TMRM and MTR/Calcein‐AM provides information about mitochondrial mass, with the advantage of immediate application in primary cultures. The rapid timescale of our observations (immediate responses to ferutinin) precedes potential autophagic processes, which typically develop over hours. This, combined with our two independent approaches showing preservation of mitochondrial mass, strongly supports our interpretation that the observed TMRM signal changes reflect genuine depolarization events rather than autophagic degradation of mitochondria.

The difference in mPTP‐dependent apoptotic thresholds for the cells studied likely arises from molecular variability in the regulation of mitochondrial permeability transition. Early studies showed greater resistance of isolated neuronal mitochondria to stimuli that cause mPTP opening compared to mitochondria from the liver, due to ultrastructural mitochondrial features (Berman Watkins and Hastings 2000). For example, the expression of proapoptotic and antiapoptotic factors can also contribute not only to the sensitivity of the cell to apoptotic signals but could also directly affect the permeability of the mitochondrial membrane (Singh Letai and Sarosiek 2019). Bax and Bcl‐2 are key mediators of apoptosis acting on the intrinsic, mitochondrial pathway of programmed cell death. The proapoptotic Bax protein oligomerizes on the outer mitochondrial membrane, compromising its integrity to facilitate the release of cytochrome c and other death enzymes that activate cell demolition. In contrast, the antiapoptotic Bcl‐2 protects cell viability by preserving mitochondrial integrity and preventing cytochrome c efflux. Consequently, the relative expression levels and balance between these factors plays a pivotal role governing apoptosis initiation (Singh Letai and Sarosiek 2019). Hence, increased expression of pore‐forming proapoptotic Bax and decreased antiapoptotic Bcl‐2 levels in fibroblasts compared to neuron‐glial culture, likely prime their mitochondria for changes in permeability (Figure 8C). It is important to note that for this study we have used a mature neuron‐glial culture with a formed network. Previous studies have shown that growing neurons have high Bax and low Bcl‐2 expression, making them more vulnerable to apoptotic stimuli, whereas mature neurons show decreased Bax expression (Hollville Romero and Deshmukh 2019).

It should be noted that this study utilized cells from different species (human fibroblasts and BT‐474 cells, rat primary neuron‐glial cultures). While the observed differences in mPTP sensitivity correlate strongly with Bax/Bcl‐2 ratios rather than species origin, potential species‐specific variations in calcium handling, mitochondrial function, or death pathway regulation cannot be completely excluded. However, the fundamental mechanisms of mitochondrial‐mediated cell death are highly conserved across mammals, and the clear correlation between cellular responses and Bax/Bcl‐2 ratios suggests that the observed differences primarily reflect cell‐type specific characteristics rather than species‐specific variations.

Cells in the various tissues are protected against undesirable triggering of apoptosis with different mechanisms. Thus, mitochondria in cells have different calcium or ROS threshold for opening of mPTP (Abramov and Duchen 2011; Gandhi et al. 2009) it regulates by found by us here the difference in the number of mitochondria with mPTP opened which induce cell death.

## Author Contributions

Experiments: Kristina A. Kritskaya, Olga A. Stelmashchuk. Conceptualization and design of experiments: Andrey Y. Abramov, Kristina A. Kritskaya. Analysis: Kristina A. Kritskaya, Olga A. Stelmashchuk. Writing: Kristina A. Kritskaya, Andrey Y. Abramov. Corrections: Andrey Y. Abramov, Kristina A. Kritskaya, and Olga A. Stelmashchuk. All authors read and approved the final manuscript.

## Conflicts of Interest

The authors declare no conflicts of interest.

## Data Availability

The data that support the findings of this study are available from the corresponding author upon reasonable request.
